# Acute kidney injury after ingestion of rhubarb: secondary oxalate nephropathy in a patient with type 1 diabetes

**DOI:** 10.1186/1471-2369-13-141

**Published:** 2012-10-30

**Authors:** Marc Albersmeyer, Robert Hilge, Angelika Schröttle, Max Weiss, Thomas Sitter, Volker Vielhauer

**Affiliations:** 1 Nephrologisches Zentrum, Medizinische Klinik und Poliklinik IV, Campus Innenstadt, Klinikum der Ludwig-Maximilians-Universität München, Ziemssenstr. 1, 80336, Munich, Germany; 2 Pathologisches Institut, Ludwig-Maximilians-Universität München, Thalkirchner Str. 36, 80337, Munich, Germany

**Keywords:** Renal failure, Oxalate, Hyperoxaluria, Rhubarb, Malabsorption, Diabetes

## Abstract

**Background:**

Oxalosis is a metabolic disorder characterized by deposition of oxalate crystals in various organs including the kidney. Whereas primary forms result from genetic defects in oxalate metabolism, secondary forms of oxalosis can result from excessive intestinal oxalate absorption or increased endogenous production, e.g. after intoxication with ethylene glycol.

**Case presentation:**

Here, we describe a case of acute crystal-induced renal failure associated with excessive ingestion of rhubarb in a type 1 diabetic with previously normal excretory renal function. Renal biopsy revealed mild mesangial sclerosis, but prominent tubular deposition of oxalate crystals in the kidney. Oxalate serum levels were increased.

**Conclusion:**

Acute secondary oxalate nephropathy due to excessive dietary intake of oxalate may lead to acute renal failure in patients with preexisting renal disease like mild diabetic nephropathy. Attention should be payed to special food behaviors when reasons for acute renal failure are explored.

## Background

Oxalosis is a metabolic disorder characterized by deposition of oxalate crystals in various organs including the kidney. Primary and secondary forms of oxalosis can be distinguished. Primary oxalosis or hyperoxaluria (PH) is caused by genetic disorders. The most common genetic defects comprise mutations in the genes encoding for alanin-glyoxylate-aminotransferase (*AGXT*) resulting in PH type 1, and glyoxylate-reductase/hydroxypyruvat-reductase (*GRHPR)* causing PH type 2. Recently, a third gene defect leading to a deficiency of 4-hydroxy-2-oxoglutarate aldolase (*HOGA1*) has been identified as the cause of PH type 3 [1]. Typically, disease manifestations occur in childhood or adolescence. Prognosis is poor, since approximately 50% of diagnosed patients progress to end-stage renal disease (ESRD). Treatment of primary forms comprises hemodialysis or peritoneal dialysis for renal failure, yet cannot prevent progression of disease as elimination of oxalate by dialysis is insufficient. Therefore, combined kidney/liver transplantation is the treatment of choice [2].

Secondary forms of hyperoxaluria may result from increased intestinal oxalate absorption (termed enteric hyperoxaluria), endogenous production, or increased dietary intake. Enteric hyperoxaluria accounts for approximately 5% of all cases of hyperoxaluria and is associated with fat malabsorption due to small bowel disease or cystic fibrosis. The suggested mechanism leading to hyperoxaluria in these cases is a net loss of calcium via unresorbed bile acids, which in turn is not available to bind oxalate in the intestinal lumen. Calcium-bound oxalate is normally excreted via the feces, whereas unbound oxalate can be absorbed via the colonic mucosa [3]. Interestingly, bariatric surgery can increase oxalate absorption in the colon and thus may be an iatrogenic cause of hyperoxaluria [4]. Hyperoxaluria can also result from ingestion of ethylene glycol, high doses of vitamin C [5], or vitamin B6 deficiency [4] that result in increased endogenous production of oxalate. Other described causes include hyperparythyreoidism and sarcoidosis [6].

Here, we describe a case of acute renal failure after excessive ingestion of rhubarb in a type 1 diabetic with previously normal excretory renal function, with tubular deposition of oxalate crystals evident on renal biopsy.

## Case presentation

The 52 year-old caucasian female patient suffered from reoccurring episodes of nausea and vomiting in the preceding days. With a known history of type 1 diabetes, the patient was admitted to clinic in a state of severe hypoglycemia. On admission an elevated serum creatinine was noted, with creatinine levels initially being 3.6 mg/dl and an estimated glomerular filtration rate (eGFR, according to the modified of diet in renal disease (MDRD) equation) of 13.3 ml/min/1.73 m^2^. Postrenal causes were excluded by renal ultrasound which did not show any signs of obstructive nephropathy or intrarenal and ureteral concrements. Since the calculated fractional excretion of urea was 40.8% and renal function did not show improvement upon intravenous volume challenge, prerenal causes appeared unlikely and the patient was transferred to the nephrology department for further evaluation. The patient denied any use of non-steroidal antirheumatic drugs (NSAR) such as diclofenac or ibuprofen, and she had not been exposed to contrast media recently.

The patient was diagnosed with diabetes type 1 several years ago and has been treated with insulin on an intensified conventional therapy (ICT) regimen. Previously, renal function was normal, with creatinine values remaining below 1.0 mg/dl, corresponding to a eGFR > 60 ml/min/1.73 m^2^ at routine consultations with her diabetologist. However, urinalysis several years ago showed a significant microalbuminuria of 100 mg/dl as tested by screeining dipstick analysis. Thus, chronic kidney disease (CKD) stage one according to “Kidney disease: Improving global outcomes” (KDIGO) had been diagnosed. The patient’s medical history comprised a severe depression. Currently, she described a stable psychiatric situation, and had discontinued the pharmacological treatment with venlafaxin several months ago. There was no history of kidney stones both in the patient or her family. In addition, there was no history of small bowel disorders. Current medication of the patient included insulin and insulin glargin, as well as ramipril, metoprolol and low-dose aspirin. This medication has been taken for several years.

Physical examination revealed a 52-year-old patient in good physical condition. Blood pressure was slightly elevated (145/76 mmHg), heart beat at regular rate and rhythm. She was breathing normally and was afebrile. There was no lymphadenopathy in the cervical, nuchal, supra- or infraclavicular regions. Skin examination was unremarkable. Mucous membranes were moist without signs of candidosis. Lung and heart auscultation were unremarkable. Jugular veins were slightly distended at 2 cm on both sides; however, no significant peripheral edema was present. Abdominal examination was unremarkable. The patient showed signs of lower leg varicosis bilaterally. Vascular status showed a lack of palpable pulses downstream of the popliteal artery on the left side, no foot ulcerations were noted. Neurological examination was unremarkable.

Laboratory testing showed a mild hyperchromic anemia (Hb 10.5 g/dl). Serum creatinine was 3.6 mg/dl, corresponding to an eGFR of 13.3 ml/min/1.73 m^2^ using the MDRD-equation (Table 1). The patient presented with hyperkalemia (5.9 mmol/l), hyperphosphatemia of 5.1 mg/dl (reference range 2.5-4.8 mg/dl) and a mild hypercalcemia as revealed by a protein-corrected calcium value of 2.55 mmol/l (reference range 2.15-2.50 mmol/l). Thyroid and liver function tests were unremarkable. Urine examination revealed a proteinuria of 3.7 g protein per g creatinine, being mostly albumin (2.6 g/g creatinine). Intact parathyroid hormone was increased to 103 pg/ml (reference range 15–65 pg/ml). Urine microscopy showed no evidence for acanthocytes, erythrocyte casts, tubular casts or crystals. Autoantibody testing revealed a slightly elevated ANA-titer of 1:120, with normal values for complement. ANCA-antibodies and anti-GBM-antibodies were not detected.

**Table 1:** Laboratory data at presentation

**Variable**	**Reference range**	**Result**
**Blood Count**		
Leukocytes (G/l)	4.0−11.0	7.9
Erythrocytes (T/)	4.20−5.10	**3.1**
Hemoglobin (g/dl)	12.0−16.0	**10.5**
Hematocrit (%)	36.0−46.00	**30.4**
Mean corpuscular volume (fl)	78.0−98.0	98
Mean corpuscular hemoglobin (pg)	26.0−32.0	**33.9**
Mean corpuscular hemoglobin concentration (g/dl)	32.0−36.0	34.6
Platelet count (G/l)	150−400	351
**Serumchemistry**		
Sodium (mmol/l)	135−145	138
Potassium (mmol/l)	3.5−5.0	**5.9**
Glucose (mg/dl)	60−100	**191**
Urea nitrogen (mg/dl)	7−23	**55**
Creatinine (mg/dl)	0.5−1.0	**3.6**
Estimated glomerular filtration rate (MDRD) (ml/min/1.73 m^2^)	≥60.0	**13.3**
Calcium (mg/dl)	2.15−2.50	**2.53**
Calcium (protein-corrected) (mmol/l)	2.15−2.50	**2.55**
Phosphorus (mg/dl)	2.5−4.8	**5.1**
C−reactive protein (mg/dl)	<0.50	0.18
Total protein (g/dl)	6.0−8.5	7.6
Carbone dioxide (mmol/l)	23.0−29.0	**22.2**
Intact parathyroid hormone (pg/ml)	15−65	**103**
**Urinalysis**		
**Screening dipstick test**		
White cells (per μl)	0-10	0
Nitrites	neg.	neg.
pH	5.0−6.0	5.5
Protein (mg/dl)	neg.	**>300**
Glucose (mg/dl)	neg.	**500**
Ketones (mg/dl)	neg.	neg.
Urobilinogen (mg/dl)	<1.0	0.2
Bilirubin	neg.	neg.
Blood (per μl)	0-5	**appr. 25**
Specific weight (g/l)	1005−1030	1025
**Sediment**		
White cells (per high-power field)	<10	1-3
Red cells (per high-power field)	<2	1-3
Epithelial cells (per high-power field)	-	**+**
Hyaline casts (per low-power field)	-	**+**
Granulated casts (per low-power field)	-	**+**
Cellular casts (per low-power field)	-	**+**
**Urinary chemistry**		
BUN (urine) (mg/dl)	700.0−1213.0	492
Creatinine (urine) (mg/dl)	30.0−220.0	79.2
Protein (urine) (mg/dl)	<15.0	**296**
Protein/creatinine ratio (mg/g)	<100.0	**3,740**
Albumin (urine) (mg/dl)	<2.0	**210**
Albumin/creatinine ratio (mg/g))	<20.0	**2,645**
α1−Microglobulin (urine) (mg/l)	<12.0	**106**
α1−Microglobulin/creatinine (mg/g)	<14.0	**133**
**Immunology**		
Monoclonal immunoglobulins		negative
Complement C3 (mg/dl)	90.0−180.0	115
Complement C4 (mg/dl)	10.0−40.0	31.7
Antinuclear antibodies (ANA)		**1:120**
Antineutrophil cytoplasmic antibodies (ANCA)		negative
Anti basal membrane antibodies (ELISA) (IU/ml)	<20.000	<20.0

Values outside the reference range are shown in bold print.

**Figure 1:**
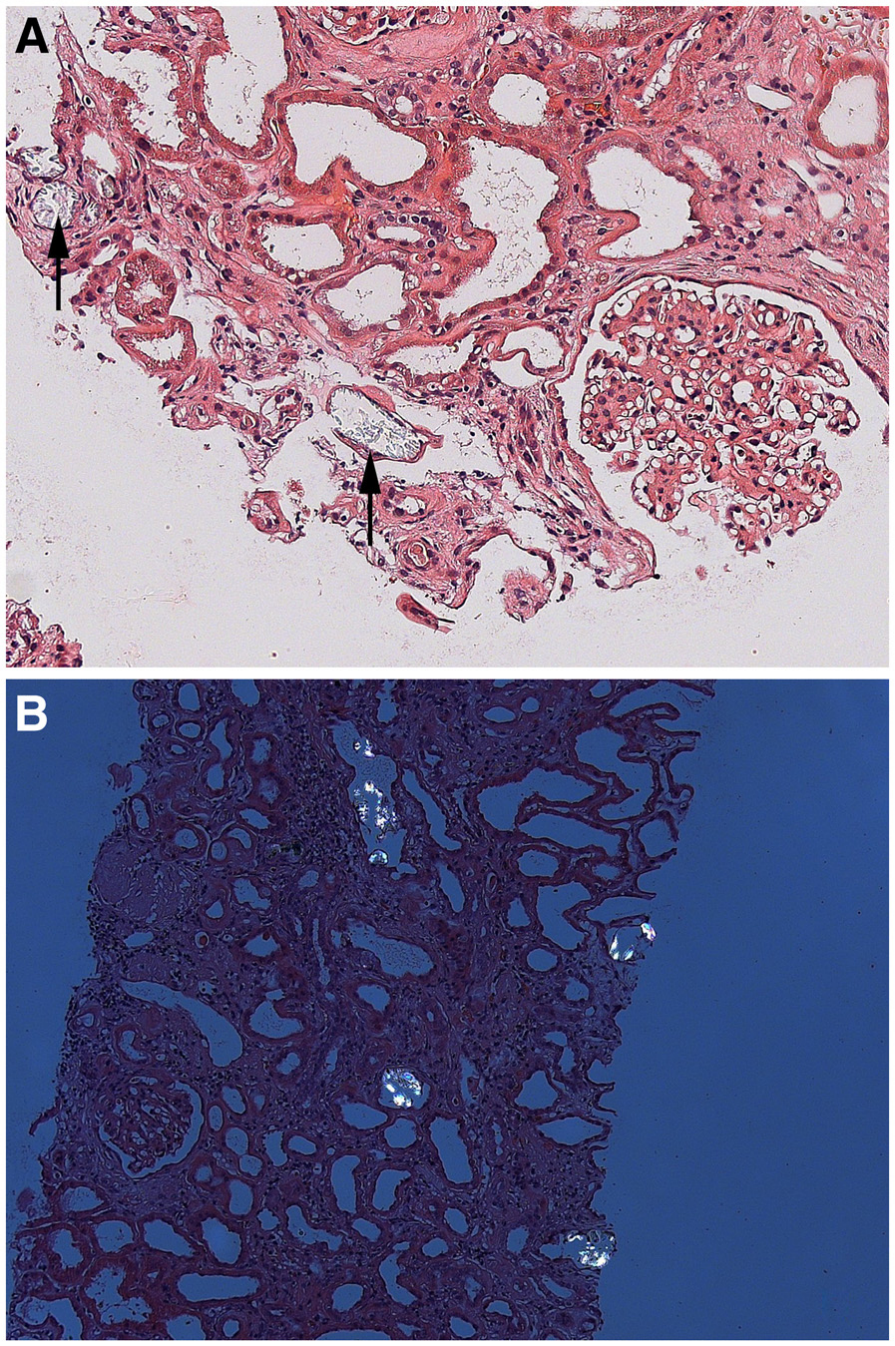
**(A) Oxalate crystals (arrows) within the renal tubular lumen in a hematoxylin eosin-stained section (original magnification x200) and (B) under polarisation light microscopy (original magnification x100).** Adjacent glomeruli show mild diabetic mesangial sclerosis.

A kidney biopsy was performed and showed mild diabetic mesangial sclerosis. Moreover, multiple birefringent crystallinous casts were found within the tubular lumen (Figure 1). No changes typical for ischemic renal failure were noted. Upon further questioning, the patient reported an increased ingestion of approximately 500 mg of rhubarb (fresh weight) per day in the last 4 weeks. Consumption of vitamin C or ethylene glycol was denied. Additional laboratory tests revealed elevated serum oxalate levels (13.75 μmol/l, reference range <6.5 μmol/l), whereas urinary excretion of oxalate, glycolate, citrate and calcium were normal (Table 2). Serum levels of folic acid, as well as vitamin B12 were within the normal range. The patient underwent an ophthalmological examination which showed no evidence for corneal of retinal oxalate deposition.

**Table 2:** 24 hour urine analysis

**Variable**	**Referance range**	**Result**
Oxalate (mmol/1,73 m^2^/d)	<0.5	0.39
Glycolate (mmol/1,73 m^2^/d)	<0.5	0.34
Citrate (mmol/d)	0.3 – 7.4	2.25
Calcium (mg/d)	<250	176

The history of high oxalate intake, the elevated serum oxalate levels, and the histological detection of intratubular birefringent crystals lead to the diagnosis of secondary oxalosis, being the most likely cause for the acute decline in the patient`s renal function. Since no established causative therapy for secondary oxalosis is available, intravenous fluid replacement was started, leading to an initial stabilization of renal function with an eGFR at 15 ml/min/1.73 m^2^. However, upon discontinuation of the intravenous fluid regimen, creatinine levels slowly rose to a maximum of 4.3 mg/dl (eGFR 10.3 ml/min/1.73 m^2^) within 2 months. Currently, the patient is treated in our outpatient clinic for end stage renal disease (ESRD), and options for fistulas are being evaluated in preparation for hemodialysis.

## Discussion

Apart from genetic forms of hyperoxaluria (PH1 to 3), hyperoxaluria and hyperoxalemia can occur upon increased dietary intake of foods that have a high oxalate content such as cocoa (mean oxalate content 700 mg/100 g fresh weight (FW) [7]), rhubarb (raw 805 mg/100 g FW, stewed 460 mg/100 g FW [7]), spinach (970 mg/100 g FW [7]), black teas (1150 mg/100 g FW [7]), beet leaves (610 mg/100 g FW [7]), and star fruit (263 mg/100 g FW [8]). Interestingly, there is some data that herbal medicines containing rhubarb might actually improve renal function, including the reduction of proteiuria, slowing progression of chronic kidney disease or ameliorating dyslipidemia [9]. Anti-inflammatory and anti-fibrotic effects are discussed. It has not been reported that regular consumption of these preparations caused secondary hyperoxaluria and renal failure. Since ascorbic acid can be metabolized to oxalate, high intake of vitamin C may also cause hyperoxaluria [5].

Several cases of acute deterioration of renal function secondary to oxalate crystal deposition have been described with a majority comprising malabsorption syndromes such as pancreatitis or gastric by-pass as well as non-renal organ transplant recipients [10-12]. Recent reports illustrated a deteriorated renal function after consumption of star fruit [13] or peanuts [14], both of which containing high amounts of oxalate. There are only few reports that described oral rhubarb ingestion as a cause of oxalate-induced kidney injury, most of which occurred in children [15].

Here, we report an adult patient with initially unexplained acute renal failure. Surprisingly, her kidney biopsy revealed deposition of disseminated crystals within the tubular lumen suggestive for calcium oxalate deposition. As no other causes of the patient’s acute renal failure could be elucidated, she was carefully questioned regarding oxalate intake. She reported an extensive consumption of rhubarb, i.e. 500 mg fresh weight of plant per day for the least 4 weeks. This resulted in an uptake of approximately 2.3 g oxalate per day [7]. In a study by Holmes et al. a dose of approximately 1 g oxalate was given to healthy subjects without evidence of acute renal injury or oxidative stress [16]. However, the minimal dose of oxalate causing death in adults is 4–5 g [15]. These cases often show typical birefringent oxalate deposition in the tubular lumen and in tubular epithelial cells [15]. To our knowledge, there is no data defining a threshold for oxalate intake that would result in nephropathy. Our patient had a known history of diabetes mellitus type 1 and chronic kidney disease stage I with microalbuminuria, but without a previous impairment of excretory renal function. We assume that in this patient a mildly injured diabetic kidney suffered a“second hit”by oxalate crystal deposition within tubuli leading to acute renal failure.

In addition, a prerenal component may have contributed to the acute kidney failure due to the initial episode of nausea and vomiting with a prolonged state of hypoglycemia. Importantly, there is evidence that reductions of GFR facilitate precipitation of calcium-oxalate crystals [17]. However, renal biopsy revealed no changes suggestive for prerenal or ischemic renal failure, but showed prominent oxalate crystal deposition. Thus, it is unlikely that dehydration was the primary cause that lead to a prerenal form of renal failure in this patient. Instead, the patient’s initial episodes of nausea and vomiting could have been symptoms of her acute renal failure.

There are few data on long term-outcomes of patients with secondary oxalate nephropathy. A retrospective analysis of twelve French patients with acute oxalate nephropathy reported ESRD in three of 12 patients after a median follow-up of 7 months [10]. Therapeutic approaches are limited and comprise increased fluid intake, probiotics [18], low-oxalate diet [19] and hemodialysis or peritoneal dialysis for ESRD [20]. There is inconclusive data considering probiotic therapy. Of note, *Oxalobacter formigenes* colonizes the human intestine and relies on the metabolism of oxalate [3]. Several reports and small studies describe a potential benefit of *Oxalobacter* treatment on lowering both plasma and urinary oxalate levels [18,21]. Even though these data appear promising, there is still uncertainty about its efficacy and correct dosing, and larger studies are needed [22].

## Conclusion

We report a rare case of acute secondary oxalate nephropathy, likely due to excessive ingestion of rhubarb in an adult with preexisting mild diabetic nephropathy, leading to crystal-induced acute renal failure. Thus, we recommend careful questioning of dietary habits or intoxications when reasons for acute renal failure are investigated. Moreover, patients with chronic renal functional impairment may be advised to avoid oral intake of excessive amounts of oxalate-containing foods such as rhubarb or spinach.

## Consent

Written informed consent was obtained from the patient for publication of this case report and any accompanying images. A copy of the written consent is available for review by the Editor-in-Chief of this journal.

## Competing interests

The authors declare that they have no competing interests.

## Authors’ contributions

MA, RH, AS, TS and VV treated the patient and provided data about the history and laboratory results. MW interpreted the kidney biopsy. MA and VV drafted the manuscript. All authors read and approved the final manuscript.

## Pre-publication history

The pre-publication history for this paper can be accessed here:

http://www.biomedcentral.com/1471-2369/13/141/prepub
